# A Virome and Proteomic Analysis of Placental Microbiota in Pregnancies with and without Fetal Growth Restriction

**DOI:** 10.3390/cells13211753

**Published:** 2024-10-23

**Authors:** Aleksandra Stupak, Maciej Kwiatek, Tomasz Gęca, Anna Kwaśniewska, Radosław Mlak, Robert Nawrot, Anna Goździcka-Józefiak, Wojciech Kwaśniewski

**Affiliations:** 1 Chair and Department of Obstetrics and Pathology of Pregnancy, Medical University of Lublin, 20-081 Lublin, Poland; maciej.kwiatek@umlub.pl (M.K.); tomasz.geca@umlub.pl (T.G.); anna.kwasniewska@umlub.pl (A.K.); 2 Body Composition Research Laboratory, Department of Preclinical Science, Medical University of Lublin, 20-059 Lublin, Poland; radoslaw.mlak@umlub.pl; 3 Department of Molecular Virology, Institute of Experimental Biology, Adam Mickiewicz University in Poznan, 61-712 Poznań, Poland; rnawrot@amu.edu.pl (R.N.); agjozef@amu.edu.pl (A.G.-J.); 4 Department of Gynecologic Oncology and Gynecology of the Medical University of Lublin, 20-081 Lublin, Poland; wojciech.kwasniewski@umlub.pl

**Keywords:** virome, proteomic analysis, microbiome, fetal growth restriction, pregnancy, placenta

## Abstract

Introduction: Metagenomic research has allowed the identification of numerous viruses present in the human body. Viruses may significantly increase the likelihood of developing intrauterine fetal growth restriction (FGR). The goal of this study was to examine and compare the virome of normal and FGR placentas using proteomic techniques. Methods: The study group of 18 women with late FGR was compared with 18 control patients with physiological pregnancy and eutrophic fetus. Proteins from the collected afterbirth placentas were isolated and examined using liquid chromatography linked to a mass spectrometer. Results: In this study, a group of 107 viral proteins were detected compared to 346 in the controls. In total, 41 proteins were common in both groups. In total, 64 proteins occurred only in the study group and indicated the presence of bacterial phages: *E. coli*, *Bacillus*, *Mediterranenean*, *Edwardsiella*, *Propionibacterium*, *Salmonella*, *Paenibaciilus* and amoebae *Mimiviridae*, *Acanthamoeba polyphaga*, *Mimivivirus*, *Pandoravirdae*, *Miroviridae*, *Pepper plant virus golden mosaic virus*, pol proteins of *HIV-1* virus, and proteins of *Pandoravirdae*, *Microviridae*, and heat shock proteins of the virus *Faustoviridae.* Out of 297 proteins found only in the control group, only 2 viral proteins occurred statistically significantly more frequently: 1/*hypothetical protein* [uncultured *Mediterranean* phage uvMED] and VP4 [*Gokushovirus* WZ-2015a]. Discussion: The detection of certain viral proteins exclusively in the control group suggests that they may play a protective role. Likewise, the proteins identified only in the study group could indicate a potentially pathogenic function. A virome study may be used to identify an early infection, evaluate its progress, and possible association with fetal growth restriction. Utilizing this technology, an individualized patient therapy is forthcoming, e.g., vaccines.

## 1. Introduction

The human body, like all metazoans (multicellular organisms including mitochondrial eukaryotes), is inhabited by numerous microorganisms that coexist with it. All microorganisms (bacteria, archaea, fungi, protists, and viruses) living in the human body constitute a specific microbiome. The community of genomes present in a specific environment is defined by the term microbiota [1].

As a holobiont, humans live in symbiosis with a multitude of microorganisms which play a significant, yet not fully understood, role in bodily functions [2,3]. A set of holobiont genomes (human genome + microorganism genomes) constitute the hologenome [4]. The term describes the combination of the host genome (humans ~23,000 genes) and the associated collective microbial genomes (>33 million genes, including 9 million unique protein-coding genes) [5,6].

One of the important components of any microbiome is the virome. Metagenomic research has allowed for the identification of numerous viruses present in the human body, the importance of which for the functioning of the body and in human health is not fully known [7].

Viruses (from the Latin word “virus”, poison) are the most ubiquitous and numerous of all evolutionary entities [8,9,10]. The virome of a healthy person consists of bacteriophages (mainly lysogenic phages), viruses that attack archaea, and viruses that infect human cells. Food containing viruses are a risk factor in the spread of viral infections. The most numerous group of viruses in humans are bacteriophages (10^15^), which affect the bacterial community [11]. They constitute a protective barrier against bacterial infections and also affect the immune system, directly inducing a humoral response or indirectly causing a non-specific immunomodulatory effect on the innate and adaptive immune response.

Nearly 100,000 sequences of human endogenous retroviruses (HERV) are present in the human genome, which constitute about 8% of the human genome [12]. HERV sequences entered the primate genome over 30 million years ago, undergoing significant changes through the accumulation of point mutations, deletions, and insertions. The human virome changes due to infections, changes in diet, climate, and individual factors such as age or immune status.

The results of numerous studies on human viromes indicate a correlation between virome fluctuations and certain diseases [13,14,15]. Based on them, it is believed that healthy people may be infected chronically or temporarily even with several types of viruses. Their importance for human health and development requires explanation. Viral infections may significantly affect the composition of other components of the microbiome and their functioning. However, we do not know whether these altered viriomes actually contribute to the development of the disease or only correlate with it [16].

On the other hand, some viruses may have protective effects, e.g., emerging evidence suggests that hepatitis G virus (HGV) may have a protective effect against human immunodeficiency virus (HIV)-related diseases [17]. Many studies indicate that it is not even the number and type of bacterial and viral infections that cause the disease, but dysbiosis. The impact of dysbiosis on the immune system is probably most critical in the period from birth to age two [18]. Accurate knowledge of the human virome will allow us to explain numerous health problems and earlier detection of a number of infections and appropriate therapy. The occurrence of endogenous retroviruses (ERVs) has been suggested to have far-reaching implications for our understanding of mammalian evolution and reproductive health. Most research in this area has focused on ERV-derived proteins that are involved in promoting cell–cell fusion and immune modulation in the placenta. ERVs also contain regulatory sequences (env (syncytin) genes) that have the potential to control placental gene expression both in terms of structural and functional needs [19]. Moreover, there is evidence that the new virus–host symbiont gains a very significant advantage compared to the uncolonized host, which remains susceptible to diseases caused by the related viruses [20].

Viruses are a poorly understood factor that significantly increases the likelihood of developing intrauterine fetal growth restrictions. Often, due to the asymptomatic course of infection in the mother, who may only be a carrier of a given pathogen, this factor is not diagnosed. Infectious factors may also deepen fetal growth inhibition that has already been initiated by another factor and affect the functioning of the immune system. Research indicates that, in fetuses with FGR, the total number of T lymphocytes and B lymphocytes is much lower than in normally developed fetuses [21]. There is also an atrophying of the thymus and reduced effectiveness of the immune response, among other factors, such as a lower activity in NK (natural killers) cells and a reduction in the expression level of antiviral proteins such as perforins and interferon. Impairment of the immune system of fetuses with FGR may result in a much greater susceptibility to TORCH syndrome.

Previous research shows that the existence of a placental barrier to infectious agents is not entirely true [22]. Viruses that overcome this barrier include viruses belonging to the *Herpesviridae* family, whose genome consists of double-stranded DNA (dsDNA). These are mainly CMV (cytomegalovirus) and HSV (herpes virus) viruses, which cause disruption in the normal development of the fetus mainly through underdevelopment of the placentas. It is estimated that about 1% of pregnant women are carriers of this virus. Cytomegalovirus infection causes the deposition of fibrinoid deposits, leading to placental insufficiency. Another virus influencing the development of FGR is HIV, which belongs to the retrovirus family and whose genome consists of two positive-stranded RNA strands.

The number of scientific reports regarding the biology of the placenta, and especially the viriome in pregnancy with fetal growth restriction, is scarce. In the work of Yoffe et al., comparing physiological and preeclampsia pregnancies, viral RNA was detected in extremely small amounts, below the threshold of statistical significance, so the viral load could not be determined [23]. The use of proteomic methods in virological research can significantly facilitate the identification of viruses in the human body.

### Aim of Research/Hypothesis

FGR may result from maternal, fetal, or placental factors and significantly increase the risks of intrauterine demise, neonatal morbidity, and mortality. In our view, the current challenge is to provide convincing evidence that a microbiome or virome exists in the placenta, which is not sterile. Rather than simply culturing microorganisms, the focus should be on demonstrating that the placenta is connected to and influenced by surrounding areas (such as the abdominal cavity, intestines, and vagina), where proteins from other organisms can be detected. The aim of the present study was to analyze and compare the virome of normal and FGR placentas using proteomic methods. In our studies, viruses present in normal and FGR placentas were identified based on the analysis of a set of virus proteins (virus proteome) present in the examined clinical material.

## 2. Materials and Methods

### 2.1. Material

This study involved women hospitalized at the Department of Obstetrics and Pathology of Pregnancy at the Medical University of Lublin between 2019 and 2021. These patients were between 32 and 36 weeks pregnant with a singleton pregnancy and late-onset FGR. Specific inclusion and exclusion criteria were outlined in the article by Stupak et al. [24].

Only Caucasian women were involved in the study: 18 pregnant and eutrophic fetus (estimated fetal weight EFW > 10th percentile, the upper limit was 90th percentile because EFW above that value is considered to be large for a gestational-age fetus (LGA)) (control group) and 18 women with late FGR identified after 32 weeks of pregnancy, according to Delphi consensus (study group) [25]. The gestational age was calculated using the latest menstrual period and first-trimester ultrasonography (based on crown-rump length-CRL). Due to significant correlation between abnormal Doppler of both the umbilical and uterine arteries (UtA) and adverse outcome of pregnancy, all women underwent a Doppler examination of those vessels [26]. During one week before the birth, Doppler measurements of the umbilical artery-free loop were taken using a Voluson E9 with RA4B 3D 4–8 MHz curvilinear probe (GE Healthcare, Buckinghamshire, UK). Then, the pulsatility index (PI) and cerebroplacental ratio (CPR) were conducted. Using standardized medical records and patient interviews, smoking, age, weight, and body mass index (BMI) at the beginning of the first trimester, pregnancy weight increase, the BMI and TORCH (toxoplasmosis, other, rubella, cytomegalovirus, herpes) were determined for the mothers. The data, including information on infants, were described in our previous work [27,28,29,30].

Material for the proteomic examination were fragments of the maternal site of the normal placentas as controls and fragments obtained from mothers with FGR. During the cesarean section (no general anesthesia) and soon after childbirth, the placentas were put in sterile containers containing ice under aseptic circumstances. The placentae were gathered and weighed. Four placental samples, each measuring 1.0 × 1.0 × 1.5 cm, were collected from different quadrants of the placenta, from approximately 3 to 4 cm away from the umbilical cord attachment site. Each sample was placed in a sterile labeled cryovial, frozen using liquid nitrogen, and stored at −80 °C for future analysis.

### 2.2. Methods

The stages of the identification of proteins using LC-ESI-MS/MS and statistical analysis is precisely presented in our previous study [24].

## 3. Results

### 3.1. Characteristics of the Control and Study Group

The results of the study were obtained from an overall total of 36 placentas taken from women with fetal growth disorders and control placentas. The clinical characteristics, along with the anthropometric measurements of mothers and their newborns, are presented in our previous study [30].

There were no statistics significant differences between the study groups in terms of age, height, fertility, BMI prior to pregnancy, and body weight before pregnancy and at delivery. One of the statistics significant difference was the greater weight gain in the control group (*p* = 0.032). In the study group, the mean pulsation index (PI) in the uterine arteries was statistically substantially greater than in the control group (*p* = 0.025), as was the PI in the arterial umbilical cord of fetuses with FGR compared to eutrophic fetuses (*p* = 0.0001). The CPR was substantially higher in the control group than in the study group (*p* = 0.0005). Women in the FGR group delivered earlier than those in the control group (*p* = 0.001). Compared to neonates from the control group, infants with hypotrophy had a lower birth weight (*p* = 0.0001), shorter body length (*p* = 0.001), and lower 1st minute Apgar score (*p* = 0.002).

### 3.2. Analysis of the Virus Proteome in the Study and Control Groups

**Figure 1:**
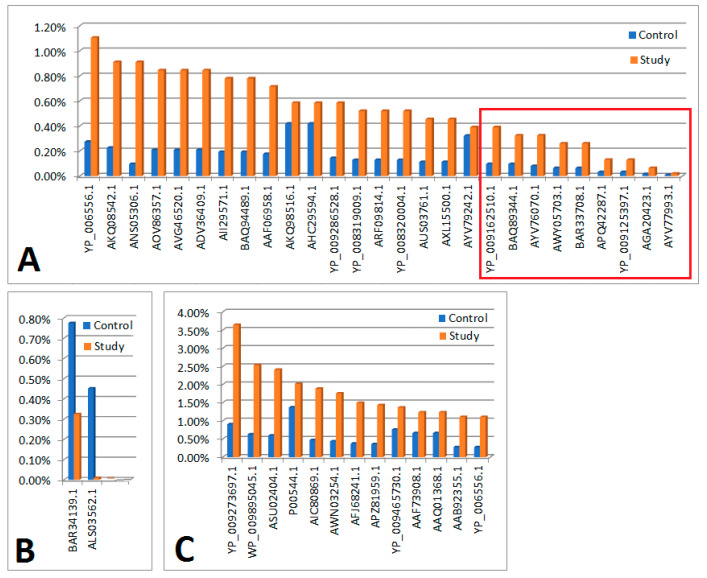
Bar graphs showing a comparison of proteins that were present in both the study and control groups. The red square indicates statistically significant results that remained significant after applying the Bonferroni correction. (**A**) Graph showing comparisons of protein content (n = 26), for which em PAI values were significantly higher in the study group compared to the control. (**B**) Graph showing comparisons of protein content (n = 2) for which em PAI values were significantly (als03562.1) or non-significantly (bar34139.1) higher in the control compared to the study group. (**C**) Graph showing comparisons of protein content (n = 13) for which em PAI values were slightly higher in the study group compared to the control.


**Figure 2:**
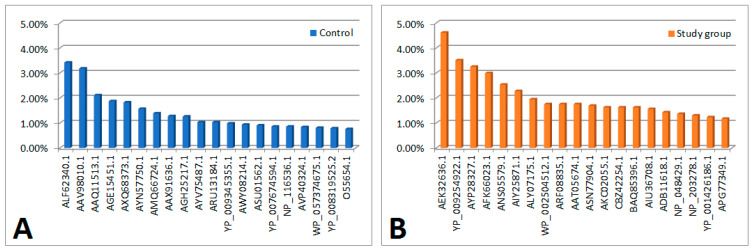
Bar charts showing the content of proteins that occurred only in material from people from the study or control groups. (**A**) Graph showing the content of proteins found only in material from controls (proteins are sorted by decreasing em PAI value) [part 1—proteins 1–20]. (**B**) A graph showing the content of proteins found only in material from people from the study group (proteins are arranged in descending order of em PAI value) [part 1—proteins 1–20].


Based on the analysis of proteinogram in material collected from people, the following results were obtained:

(a) In the study group (n = 18), 107 viral proteins were detected (with a cut-off point of ns prot score ≥ 36). Of these, 2 proteins had a 0<em>PAI value—they were not included in further analyses. Of the remaining 105 proteins, 41 were also present in the material from the control group (differences in their content in individual materials are presented in Figure 1). The remaining 64 proteins occurred only in the material collected from people from the study group (their content in the material is shown in Figure 2B).

(b) In the control group (n = 18), 346 viral proteins were detected (with a cut-off point of ns prot score ≥ 36). Of these, 8 proteins had a 0<em>PAI value—they were not included in further analyses. Of the remaining 338 proteins, 41 were also present in the material from people from the study group (differences in their content in individual materials are presented in Figure 1). The remaining 297 proteins occurred only in the material collected from the control group (their content in the material is shown in Figure 2A).

Study group: In total, 64 viral proteins found only in the this group are mainly bacterial phages: E. coli, Bacillus0, Mediterranenean, Edwardsiella, Propionibacterium, Salmonella, Paenibaciilus, and mimivirus: Acanthamoeba polyphaga mimivirus, Pepper golden mosaic virus, HIV-1 pol proteins, Pandoravirus, Microviridae proteins, and heat shock proteins of the virus Faunusvirus. 

Control group: Out of 297 proteins found only in the control group, only 2 viral proteins occurred statistically significantly more frequently: 1/hypothetical protein [uncultured Mediterranean phage uvMED] and VP4 [Gokushovirus WZ-2015a].

**Legend**

**Figure 1A**
In the order as in the figure
**Significant differences in the control and test material in terms of protein content determined on the basis of em PAI**
YP_006556.1Ppp [Escherichia virus P1]AKQ08542.1putative HNH endonuclease [Bacillus phage PBC2]ANS05306.1hypothetical protein [uncultured Mediterranean phage]AOV86357.1putative replication protein VP4 [uncultured virus]AVG46520.1ankyrin repeat protein [Acanthamoeba polyphaga mimivirus]ADV36409.1tail fiber [Edwardsiella phage eiAU]AII29571.1exonuclease [Propionibacterium phage PHL117M01]BAQ94489.1minor capsid protein 10 [uncultured Mediterranean phage uvMED]AAF06958.1replication initiation protein [Pepper golden mosaic virus-[CR]]AKQ98516.1pol protein, partial [Human immunodeficiency virus 1]AHC29594.1pol protein, partial [Human immunodeficiency virus 1]YP_009286528.1head vertex protein II [Salmonella phage vB_SnwM_CGG4-1]YP_008319009.1hypothetical protein pdul_cds_330 [Pandoravirus dulcis]ARF09814.1DEAD/SNF2-like helicase [Indivirus ILV1]YP_008320004.1hypothetical protein pdul_cds_1056 [Pandoravirus dulcis]AUS03761.1terminase large subunit [Paenibacillus phage Tadhana]AXL15500.1major capsid protein [Microviridae sp.]AYV79242.1heat shock protein [Faunusvirus sp.]
**Significant differences in the control and test material in terms of protein content determined on the basis of em PAI (maintaining significance after applying the Bonferoni correction)**
YP_009162510.1DEAD-like helicases superfamily D11 [Salmon gill poxvirus]BAQ89344.1hypothetical protein [uncultured Mediterranean phage uvMED]AYV76070.1hsp82-like protein [Terrestrivirus sp.]AWY05703.1tape measure protein [Microbacterium phage Percival]BAR33708.1internal virion protein D [uncultured Mediterranean phage uvMED]APQ42287.1tape measure protein [Mycobacterium phage Rich]YP_009125397.1tapemeasure [Mycobacterium phage Sparky]AGA20423.1polyprotein [Deformed wing virus]AYV77993.1hypothetical protein Edafosvirus3_71 [Edafosvirus sp.]
**Figure 1B**


**Significant or *negligible* differences in the control and test material in terms of protein content determined on the basis of em PAI**
BAR34139.1hypothetical protein [uncultured Mediterranean phage uvMED]ALS03562.1VP4 [Gokushovirus WZ-2015a]
**Figure 1C**


**There were no significant differences in the control and test material in terms of protein content determined on the basis of em PAI**
YP_009273697.1hypothetical protein PAEP54_00150 [Pseudomonas phage YMC11/07/P54_PAE_BP]WP_009895045.1MULTISPECIES: hypothetical protein [Clostridiales]ASU02404.1hypothetical protein P1301_0041 [Bacteriophage T5-like chee130_1]P00544.1RecName: Full=Tyrosine-protein kinase transforming protein FgrAIC80869.1Vpr [Simian immunodeficiency virus]AWN03254.1HNH endonuclease [Microbacterium phage Camille]AFJ68241.1non-structural protein 4 [Rotavirus A]APZ81959.1hypothetical protein EFP01_032 [Enterococcus phage EFP01]YP_009465730.1hypothetical protein DSLPV1_013 [Dishui lake phycodnavirus 1]AAF73908.1polyprotein, partial [Bovine viral diarrhea virus-1 strain CP821]AAQ01368.1glycoprotein, partial [Human metapneumovirus]AAB92355.1nonstructural protein P125-2, partial [Bovine viral diarrhea virus 1]

**Legend**

**There were no significant differences in the control and test material in terms of protein content determined on the basis of em PAI**

**Figure 2A**
In the order as in the figureALF62340.1histone 4, partial [Cotesia sesamiae bracovirus]AAV98010.1hypothetical protein ORF3006 [Cotesia plutellae polydnavirus]AAQ11513.1fusion, partial [Avian avulavirus 1]AGE15451.1p5 [Sweet potato chlorotic stunt virus]AXQ68373.1hypothetical protein CcrBL10_gp169c [Caulobacter phage CcrBL10]AYN57750.1hypothetical protein PBI_DRMANHATTAN_30 [Arthrobacter phage DrManhattan]AMQ66724.1hypothetical protein [Bacillus phage Mgbh1]AAX91636.1ORF097 [Staphylococcus virus 71]AGH25217.1hypothetical protein kp_75 [Escherichia virus KP26]AYV75487.1GTP-binding protein YPTC1 [Terrestrivirus sp.]ARU13184.1Cro-like repressor [Streptococcus phage P0095]YP_009345355.1ubiquitin [Noumeavirus]AWY08214.1hypothetical protein [Klebsiella phage ZCKP1]ASU01562.1hypothetical protein P24_0109 [Bacteriophage T5-like chee24]YP_007674594.1hypothetical protein SWZG_00236 [Synechococcus phage S-SKS1]NP_116536.1hypothetical protein BK5-Tp44 [Lactococcus phage BK5-T]AVP40324.1hypothetical protein [Staphylococcus phage phiSA_BS1]WP_057374675.1winged helix-turn-helix domain-containing protein [Propionibacterium freudenreichii]YP_008319525.2Histone H2B domain [Pandoravirus dulcis]O55654.1RecName: Full=Early E3B 10.4 kDa protein; Flags: Precursor
**Figure 2B**

AEK32636.1hypothetical protein GEORGE_68 [Mycobacterium virus George]YP_009254922.1hypothetical protein [Tokyovirus A1]AYP28327.1hypothetical protein 3M_071 [Serratia phage vB_SmaA_3M]AFK66023.1hypothetical protein OMVG_00018 [Ostreococcus lucimarinus virus OlV3]ANS05579.1SET domain containing protein [uncultured Mediterranean phage]AIY25871.1histone H4, partial [Cotesia sesamiae]ALY07175.1hypothetical protein VmeM32_00189 [Vibrio phage vB_VmeM-32]WP_002504512.1MULTISPECIES: hypothetical protein [Staphylococcus]ARF08835.1ubiquitin-conjugating enzyme E2 [Catovirus CTV1]AAT05674.1envelope glycoprotein, partial [Human immunodeficiency virus 1]ASN77904.1hypothetical protein [Grapevine virus H]AKC02055.1hypothetical protein [Cyprinid herpes virus 2]CBZ42254.1hypothetical protein [Campylobacter virus CP81]BAQ85396.1Lysozyme family protein (zliS) [uncultured Mediterranean phage uvMED]AIU36708.1ORF62 [Cydia pomonella granulovirus]ADB11618.1M70R, partial [Myxoma virus]NP_048429.1hypothetical protein [Paramecium bursaria Chlorella virus 1]NP_203278.1LEF-7 [Epiphyas postvittana nucleopolyhedrovirus]YP_001426186.1hypothetical protein FR483_n554L [Paramecium bursaria Chlorella virus FR483]APG77349.1M protein [Xinzhou nematode virus 6]

## 4. Discussion

The presence of numerous viral proteins in the material from the control, which are not present in the material obtained from people from the study group, may indicate their protective role. Similarly, the presence of proteins found only in material obtained from people in the study group may indicate their potentially pathogenic nature.

However, we must remember that the protein content in the tested sample does not necessarily correspond to the presence of a viral infection, due to the variable expression of these proteins depending on their type and needs.

The set of viruses present in various anatomical locations of the human body constitutes the human viriome and is considered to be a component of the human microbiome. The qualitative and quantitative composition of the human virome is not precisely known. At the same time, the origin of the viruses themselves is controversial. There are fascinating hypotheses stating that they could have arisen even before the LUCA cell, the last common universal ancestor of all three domains of life on Earth: Eukarya, Bacteria, and Archaea [31]. 

The Human Microbiome Project (HMP) launched in 2007 by The National Institute of Health enabled the characterization and understanding of the importance not only of microbiomes for human health and disease, but also contributed new data on the virome [32]. This project mainly analyzed eukaryotic viruses containing a double-stranded DNA (dsDNA) genome. Looking at metagenomic sequence data generated using HMP, the study detected an average of 5.5 types of viruses in each person and noted high interpersonal diversity [33].

The microbiome is very dynamic during pregnancy due to the influence of several host and environmental factors, including mode of delivery, diet, and exposure to antibiotics [34]. During the period of formation of the baby’s microbiome, all of the early interactions between microbes and humans seem to shape the systemic immune system of childhood and even adult life [35]. The abovementioned studies of the human viriome are relatively limited by the lack of common markers for viruses, heterogeneity of virome components, low biomass sample acquisition, interference by host DNA background, and the lack of standard computational tools for virome analysis and an exponentially growing virome database. However, the results of virus research so far suggest that viruses may have both beneficial and harmful effects on human health, depending on their interactions with the host and other viruses and bacteria, i.e., components of the microbiome.

Bacteriophages are mobile genetic elements that play a pivotal role in horizontal gene transfer, specifically facilitating the translocation of virulence factors between bacterial populations. The high genetic diversity and heterogeneity of both the microbiome and phages enable the continuous adaptation of the latter to new, usually insensitive bacterial hosts. This process fosters bacterial diversification and drives the constant evolution of new genotypes, potentially including novel pathogens. Ingesting bacteriophages through the diet leads to the massive destruction of bacteria in the intestines. This, in turn, leads to the release of large amounts of bacterial DNA into circulation and to an increase in the pathogen-associated molecular pattern (PAMP) [36].

Phages and bacteriophages are increasingly considered to be an alternative to antibiotic therapy in the face of growing antibiotic resistance [37]. These bacterial viruses typically have narrow antimicrobial host ranges, are self-limiting, and are unable to infect human cells. These features make them an attractive solution for treating bacteria and infections. The use of phage therapy in humans is particularly challenging during pregnancy and the perinatal period, especially for the control of *S. agalactiae*. Phage therapy has the potential to address issues related to microbiome dysbiosis as well as to reduce antibiotic resistance.

In our studies, the dominant proteins in the study group were proteins indicating the presence of viruses and/or bacterial phages in the tested sample: *E. coli*, *Bacillus*, *Mediterranenean*, *Edwardsiella*, *Propionibacterium*, *Salmonella*, *Paenibaciilus* and amoebae *Mimiviridae*, *Acanthamoeba polyphaga*, *Mimivivirus*, *Pandoravirdae*, *Miroviridae*, *Pepper plant virus golden mosaic virus*, pol proteins of *HIV-1* virus, and proteins of *Pandoravirdae*, *Microviridae*, and heat shock proteins of the virus *Faustoviridae.*

However, in the control group, only two viral proteins occurred statistically significantly more frequently: phage protein 1/*hypothetical protein* [uncultured *Mediterranean* phage uvMED] and VP4 [and *Gokushovirus* WZ-2015a].

The literature review regarding phages and placenta is very sparse. In vitro work by researchers from China on bactriophagous *P_IZ_ SAE-01E2 Salmonella entrica Serovar Abortusequi* in mice showed that this bacteriophage has the potential to block abortions in mice caused by *Salmonella enetrica Serovar Abortusequi* [38].

Particularly noteworthy is the presence of nuclear-cytoplasmic proteins of large DNA viruses—NCLDV [39]. One of the proteins identified was a viral protein Acanthamoeba polyphaga mimivirus, the first large giant virus described in 2003, with a 1.2 Mb genome that encodes 979 proteins, including central elements of the translation apparatus.

NCLDV viruses were isolated as a taxon in 2001 [40]. In our research, we identified proteins in the placental proteome, and in the proteinogram of viruses, we detected *Mimivirdae*, *Faustoviridae*, and *Pandoraviridae*. It is difficult to explain the presence of viruses infecting amoebae in the human placenta, and it seems that although amoeba infections are not very common in our population, there can only be one way for amoebae to get to the placenta and to the amniotic fluid—the oral route. Most giant viral genomes were obtained from large-scale metagenomic sequencing projects covering aquatic ecosystems (e.g., oceans, swimming pools, lakes, and cooling wastewater treatment plants) and from forest soils [41,42,43,44,45,46].

Mimivirus and Acanthamoeba infections castellanii, like most other viral infections, cause cytopathic effects (CPE) [47]. Research by Goyal et al. showed that mimivirus infections of Acannthamoeba cells castellanii cause retractions of acanthopodia and a depolymerization of the host actin filament network, as well as characteristic tubulin cleavage, a phenomenon not previously reported in the case of any intracellular pathogens. According to the abovementioned researchers, the cleavage of amoeba tubulin during Mimivirus infection is a post-replication event and Mimivirus infection leads to cell disruption and the release of the virus. Researchers hypothesized that tubulin cleavage, along with actin depolymerization during the later stages of Mimivirus assembly, is necessary for cell lysis due to apoptotic /necrotic cell death.

Pepper golden mosaic virus-[CR] probably also entered the placenta through the oral route. The mosaic virus is not harmful to humans nor pets, since the virus is specific to plants.

It should be emphasized that some microorganisms have the ability to reside and replicate within the range of free-living amoebae (FLA-*range of free-living amoebae*), which indicates the possibility of a wide environmental occurrence. The ability of *amoebae-resistant microbes* (ARM) provides them with the advantage of transport within the environment by forming permanent cysts; FLA protects the ARM against disinfectants such as chlorine [48,49]. The ubiquitous presence of FLA in soil, air, animals, plants, and water facilitates transport in the drinking water system [50]. Such microorganisms include, for example, new pathogens such as *Waddlia chondrophila*, which is an obligate intracellular bacterium that was initially isolated in a bovine fetus but which is also associated with unfavorable pregnancy outcomes and infertility in women with tubal factor infertility [51,52,53]. *W. chondrophila* has been detected in samples collected from children with respiratory infections and in people with community-acquired pneumonia [54,55]. Unlike the well-known *Chlamydia trachomatis*, which is mainly spread by sexual contact, the routes of transmission of *W. chondrophila* include consumption of milk and uncooked meat, as well as contact with animals [56].

It is well-documented that pathogens can be transmitted from mother to fetus through the placenta. However, metagenomic analysis of placental samples has detected only a small number of viral DNA sequences, such as those from human herpesviruses [57]. For some researchers, this implies that DNA virus infections in the placenta are rare, supporting the idea that the placenta is largely pathogen-free or lacks a distinct microbiome of commensal bacteria [58]. In contrast, our study utilized proteomic methods, revealing novel findings that may not have been identified through metagenomic approaches alone.

Thus, the presence of incomplete viral elements in host genomes are not simply the remnants of past viral infection and disease (virus sweeps), but should be considered as the savior of the host’s lineage by providing the capacity for self-regulation and persistence of viruses that can still threaten related species [20].

Clinical Implications: The findings of this study may have important implications for the management of FGR, potentially altering clinical practice beyond the focus on diagnosis alone. While our research team has contributed several articles on this subject, further investigation is essential to validate these findings and to identify additional proteomic markers associated with FGR. Looking ahead, future directions could include developing a straightforward diagnostic tool, such as a maternal blood test, microbiological swab, or saliva test, to detect viral or bacterial proteomes that may contribute to FGR, allowing for timely intervention. Another promising avenue would be the development of a vaccine targeting the most prevalent, yet unidentified, virus implicated in FGR, thereby enabling preventive strategies against the condition.

Conclusion: Given research findings to date, there is a clear need to further understand how the microbiome influences viral infection and whether the influence of the microbiota on host immunity plays a role in viral infection, especially during pregnancy. Many questions remain regarding the mechanisms of both the direct and indirect effects of viral infections on the microbiota of the placenta.

## Data Availability

The data presented in this study are available on request from the corresponding author.
